# A DELPHI STUDY TO IDENTIFY KEY GAIT PATTERNS AND THEIR POTENTIAL CAUSES IN PEOPLE WITH MULTIPLE SCLEROSIS

**DOI:** 10.2340/jrm.v57.42556

**Published:** 2025-06-03

**Authors:** Sjoerd T. TIMMERMANS, Marjolein M. VAN DER KROGT, Marc B. RIETBERG, Heleen BECKERMAN, Vincent DE GROOT

**Affiliations:** 1 Amsterdam UMC location Vrije Universiteit Amsterdam, Rehabilitation Medicine, Amsterdam, The Netherlands; 2 Amsterdam Movement Sciences, Rehabilitation & Development, Amsterdam, The Netherlands; 3 MS Center Amsterdam, Amsterdam UMC location Vrije Universiteit Amsterdam, The Netherlands

**Keywords:** consensus, Delphi technique, gait, multiple sclerosis

## Abstract

**Objective:**

This study aims to identify characteristic gait patterns in people with multiple sclerosis, to describe their key characteristics, and to identify their potential underlying causes.

**Design:**

a 3-round Delphi study.

**Participants:**

An international panel of 20 experts, including physiotherapists, a neurologist, rehabilitation physicians, biomechanical engineers, and movement scientists with expertise in multiple sclerosis or gait analysis.

**Methods:**

A comprehensive list of gait characteristics and underlying impairments was compiled and analysed to identify common gait patterns and their primary features and potential causes. Consensus was defined as 67% agreement.

**Results:**

Consensus was reached on 6 gait patterns in multiple sclerosis: (*i*) drop foot; (*ii*) insufficient push-off; (*iii*) stiff knee during swing; (*iv*) knee hyperextension during stance; (*i*) knee flexion in midstance; and (*vi*) enhanced gait variability. At least 69% agreement was achieved on the naming of the final gait patterns, their key characteristics, and the potential causes of each pattern.

**Conclusion:**

Consensus was achieved on 6 gait patterns, their characteristics, and potential underlying causes. The identification of these gait patterns may support clinical decision-making regarding diagnostic and treatment measures, and deepen understanding of impairments that underlie walking problems in people with multiple sclerosis.

## LAY ABSTRACT

Around 60% of people with multiple sclerosis develop walking difficulties within 10 years of diagnosis. However, healthcare professionals may not always agree on how to diagnose or treat these problems, which can make treatment less effective. This study aimed to identify common walking patterns in people with multiple sclerosis and gather potential underlying causes of these patterns. We asked 20 experts from around the world – including physiotherapists and physicians – to review and anonymously discuss different types of walking difficulties in multiple sclerosis. After several rounds of discussion, they agreed on 6 main types of walking problems, including issues such as difficulty lifting the foot or leg and problems with balance and coordination. Understanding these common walking patterns and their potential causes can help healthcare professionals make better assessment and treatment decisions. This work is important for improving care and providing more personalized treatment for people with MS who have trouble walking.

A large percentage of people with MS (PwMS) experience problems with walking within 10 years of disease onset (1, 2). This leads to a decreased quality of life and might require dependency on walking aids and on other people to retain the ability to walk (3). Gait disorders in PwMS can be caused by diverse and interacting neurological impairments in various neurological systems such as motor function, coordination and sensory function, and ranges from simple to complex aetiology (4). Balance problems, spasticity, muscle weakness, changes in motor control, and walking-induced fatigue are common impairments contributing to gait disorders in PwMS (4, 5). Furthermore, during disease progression the gait impairments can worsen over time (6). Problems secondary to MS, such as physical deconditioning, comorbidities (e.g., arthritic conditions and over-use injuries) and ageing can further impact gait. Furthermore, psychological factors could also impact gait, including fear of falling (7). Because of the broad range of causes, it is important to adequately diagnose gait impairments in PwMS and help guide specific treatment recommendations to improve function.

There are various diagnostic measures to analyse gait impairments, which include, but are not limited to, performance-based walking tests (8), observation of spatiotemporal gait parameters (9), instrumented gait analysis (10), physical examination, and patient-reported outcome measures (11). Currently, 3D gait analysis is the gold standard to analyse gait abnormalities (12). However, not every clinical centre or community health service has access to 3D gait analysis and the large amount of gait data obtained requires specialized services and interpretation, which limits widespread clinical use.

There are currently no worldwide guidelines on the diagnosis and treatment of gait disorders in PwMS. A recent review of gait patterns in PwMS showed that knee flexion in swing phase, ankle dorsiflexion at initial contact, and ankle plantar flexion during pre-swing are often decreased in PwMS (13). Spatiotemporal changes in PwMS have been described clearly, including decreased walking speed, decreased step length, and increased step width (14). Currently, a clear consensus on the nomenclature to best describe common gait patterns in clinical settings is lacking. In addition, the heterogeneity in aetiology complicates describing typical gait patterns in PwMS.

The large variety of terminology used may hamper the understanding of gait abnormalities in PwMS. Practice variation in diagnosing and treating gait disorders may impede (optimal) patient treatment. This practice variation between different clinicians, centres, and countries could be reduced by clearly defined gait patterns in PwMS. In stroke, Parkinson’s and paediatric cerebral palsy general gait subtypes have been helpful in clinical decision-making (15–20). Gait characterization could serve similar purposes in PwMS. Furthermore, understanding the factors contributing to gait impairments in PwMS, and identifying these factors adequately with a valid measurement framework, can help attain better understanding and standardization of appropriate treatment options. Therefore, the objective of this study is to identify common gait patterns that are typical of PwMS, to describe their key characteristics, and to assess their potential underlying causes.

## GENERAL METHODS

### Participants and design

To gather expertise, an online international Delphi study was performed (18). In an iterative process, structured questionnaires were sent out in 3 rounds to obtain consensus. This online method was chosen to involve international experts as participants and to combine as much worldwide knowledge as possible (21–23).

Invitees needed to be internationally recognized experts with either clinical or scientifically relevant experience within the fields of MS rehabilitation or gait analysis. They were working in an MS centre, leader of an MS research group, or worked with MS patients or with gait analysis in clinical care, ideally had internationally published (in English) on MS and gait in the last 5 years, and were proficient in English. Invitees were selected from among known experts and collaborators, as well as based on a PubMed search for top-publishing authors in the field of gait analysis in MS. Invitations were sent out to 40 potential participants. All invitees received an invitation letter that consisted of a general introduction concerning gait problems in MS, the primary study objective, information on the qualitative process of a Delphi study, the layout of the Delphi rounds that were planned, what we asked of participants, and what the study would deliver for the participants (see Appendix S1 – invitation letter and information). Participants who completed at least 2 of the 3 rounds were listed as collaborators for the scientific publication.

The invitees included physiotherapists, neurologists, rehabilitation physicians, biomechanical engineers, and movement scientists. Additionally, we asked invitees to recommend colleagues who fulfilled the inclusion criteria.

Participation was anonymous for the participants. Non-responders for a particular round were still invited to participate in subsequent rounds.

For sufficient reliability of consensus, at least 6 participants should be included, with no substantial increase in reliability above 12 participants (24–26). Therefore, a mixed group of at least 15 international experts was deemed sufficient, including different perspectives (e.g., both clinical and research, and both practical and theoretical). For the online Delphi questionnaire rounds Survalyzer (Survalyzer AG, Zurich, Switzerland) was used to send out questionnaires in English. To encourage participation, invitations to every round were reiterated at least twice. This study was not registered prospectively. Consent was implied if invitees responded positively.

The results of each questionnaire were analysed anonymously, meaning that all identifying participant information was removed from the answers provided before analysis. Agreement of above 67% (2/3) was considered adequate consensus (27), meaning that 67% or more of the participants considered the item relevant for the gait pattern. A cutoff of 67% was chosen as this reflected a majority agreement without being too strict and to not dismiss potentially useful insights. Agreement below 30% was considered clear consensus concerning non-relevant issues, meaning that a clear majority considered this item not relevant for the gait pattern. Agreement between 30% and 67% was seen as “some” consensus. Results with some consensus were resubmitted to the group in the next round, to further differentiate between clearly relevant or clearly non-relevant issues when possible. Data were collected between August 2021 and October 2022.

### Composition of research team and participants

The research team consisted of 5 people, with 2 rehabilitation physicians (STT and VG), a physiotherapist/movement scientist (MBR), a movement scientist (MMK), and a clinical epidemiologist with a background in physiotherapy (HB). The entire research team was involved in MS research, with STT, VG, and MBR also involved in clinical work with PwMS. The research team gathered, analysed, encapsulated, and fed back to the participants the information from every round.

Each of the 3 Delphi rounds is described in more detail below, including aim, methods, and results for every round.

## ROUND-SPECIFIC METHODS AND RESULTS

### Round I (aim, methods, and results)

Twenty invitees fulfilling the inclusion criteria responded positively and participated. The demographics of the 20 people who agreed to participate are presented in Table I. All participants were included as collaborators in the current study. Round I focused on gathering as much information as possible about gait patterns seen in PwMS and the underlying impairments. Information was collected using 2 open questions, specifically:

**Table I:** Demographics of the 20 participants in the Delphi study

	*n* (%)
*Country of residence*	
Italy	5 (25%)
Netherlands	2 (10%)
Belgium	2 (10%)
Canada	2 (10%)
Australia	2 (10%)
Ireland	1 (5%)
Sweden	1 (5%)
Switzerland	1 (5%)
Spain	1 (5%)
Israel	1 (5%)
United States of America	1 (5%)
United Kingdom	1 (5%)
*Primary occupation*	
MS researcher	8 (40%)
Physiotherapist	6 (30%)
Rehabilitation physician	2 (10%)
Biomechanical engineer	2 (10%)
Neurologist	1 (5%)
Clinical scientist	1 (5%)
*Setting of work with MS patients (multiple answers possible)*	
Research	20 (100%)
Clinical work	11 (55%)
Management	2 (10%)
Lifestyle education/chronic disease management	1 (5%)

1. “Which gait patterns do you (commonly) observe in patients with MS in your consultation room or gait lab, and what are the specific characteristics of these patterns?”
2. “Which impairments might, in your opinion, be responsible for these gait patterns in MS?”

The response rate of the first round was 100%. All answers concerning gait patterns and impairments were grouped and divided based on common themes and characteristics. For example, all answers involving foot drop, drop foot, or flat foot were pooled.

The most frequently used gait pattern terms were: drop foot gait, hemiparetic gait, ataxic gait, spastic gait, sensory ataxia, crouch gait, and unstable gait. The most commonly used phrases for underlying impairments were weakness, paresis, spasticity, balance impairment or balance disorder, sensory problems, and fatigue. Multiple impairments were listed that could not be linked to a single gait pattern in PwMS, but had impact on walking in general, e.g., gait speed, endurance, fatigability, and loss of dynamic gait capacity (i.e., the capacity to adapt gait to perturbations).

The results of the first Delphi round led to 10 gait patterns: (*i*) drop foot, (*ii*) stiff knee, (*iii*) knee flexion, (*iv*) knee hyperextension, (*v*) insufficient push-off, (*vi*) disturbed balance, (*vii*) somatosensory, (*viii*) ataxic, (*ix*) spastic, and (*x*) motor fatigability. These gait patterns were based on or directly taken from the answers given by the participants. The term drop foot was used by 12 participants, so adapting this to a specific gait pattern was logical. Ataxic gait was mentioned by 10 participants. The term hemiparetic gait was often given (9 times), but there was major overlap with both insufficient push-off pattern and drop foot pattern. Furthermore, the term hemiparetic was not deemed specific enough as a separate gait pattern as it can involve different characteristics and refers mostly to a paresis on 1 side of the body. So, ultimately, hemiparetic gait was not added as a separate gait pattern. In round I, spasticity was mentioned by 11 participants as playing a role in gait disorders in PwMS, and therefore a “spastic gait pattern” was included as well. To ensure that all answers were well presented and summarized, the grouped data were fed back to the participants in the following Delphi round for verification.

### Round II (aim, methods, and results)

Round II expanded on the possible gait patterns seen in PwMS and the accompanying impairments. The goal was to gather feedback on the proposed gait patterns and to allow participants to agree or disagree on all gait patterns, characteristics, and proposed terminology. The 10 identified gait patterns from round I were presented, including naming, kinematic and kinetic deviations, spatiotemporal deviations, and possible causes. It was also asked which term was preferred to describe the different combined gait characteristics. Options were “pattern,” “complex”, or “gait”. See also Appendix S1 – round-specific instructions.

The questions in round II comprised the following:

- The naming of the pattern, with answer options including: (*i*) the naming is correct; (*ii*) the pattern is correct, but I would use different wording; or (*iii*) the inclusion of this pattern is incorrect.
- The kinematic and kinetic deviations of this pattern, with answer options including: (*i*) these are correct; (*ii*) these are correct, but I would prefer different wording; (*iii*) these are not correct (and why); and (*iv*) in my opinion, this list is incomplete. I would add…
- The spatiotemporal deviations of this pattern, with answer options including: (*i*) these are correct; (*ii*) these are correct, but I would prefer different wording; (*iii*) these are not correct (and why); and (*iv*) in my opinion, this list is incomplete. I would add…
- The causes of this pattern, with answer options including (*i*) these are correct; (*ii*) these are correct, but I would prefer different wording; (*iii*) these are not correct (and why); and (*iv*) in my opinion, this list is incomplete. I would add…

The response rate of the second round was 85% (*n* = 17). The term to describe the different combined gait characteristics was “pattern”, with a consensus of 67%. Consensus on the naming of 8 out of 10 gait patterns was reached. For kinematic and kinetic characteristics only the spastic gait pattern reached consensus. For spatiotemporal parameters, 5 out of 10 gait patterns attained consensus and 4 out of 10 did so regarding the causes. For the complete consensus rates see Table II.

**Table II:** Consensus rates for the first 10 patterns, including kinematic, kinetic, spatiotemporal deviations and possible causes

Item	Pattern		Kine(ma)tic deviations			Spatiotemporal deviations			Possible causes		
	*Correct*	*Different wording*	*Correct*	*Incomplete*	*Different wording*	*Correct*	*Incomplete*	*Different wording*	*Correct*	*Incomplete*	*Different wording*
Drop foot	89%	11%	44%	33%	6%	56%	22%	6%	50%	0%	17%
Stiff knee	73%	20%	53%	27%	13%	53%	7%	0%	80%	20%	7%
Knee flexion	73%	20%	60%	7%	13%	87%	13%	0%	87%	20%	0%
Knee hyperextension	86%	7%	50%	21%	7%	71%	14%	0%	36%	43%	0%
Insufficient push-off	83%	0%	62%	0%	0%	69%	8%	0%	69%	15%	0%
Disturbed balance	54%	31%	31%	38%	0%	69%	8%	0%	62%	31%	0%
Somatosensory	39%	15%	38%	23%	0%	46%	15%	0%	54%	15%	0%
Ataxic	85%	0%	54%	8%	0%	54%	23%	0%	46%	23%	0%
Spastic	75%	17%	67%	25%	0%	58%	25%	0%	75%	8%	0%
Motor fatigability	83%	0%	50%	0%	0%	67%	8%	0%	67%	8%	0%

To be able to better unify the given answers from round II we opted for the use of pre-existing kinematic and kinetic gait terminology (28). After sorting all the information from rounds I and II we “translated”’ answers, when applicable, to consistent kinematic and kinetic terminology (e.g., “increased plantar flexion velocity in loading response” instead of “foot flat”). The intention was to use terminology that can be universally understood by all healthcare professionals and researchers, and to describe gait characteristics unambiguously and as clearly as possible.

Based on the participants’ feedback from round II, it was decided to combine the “somatosensory” and “reduced balance control” gait patterns with the “ataxic” gait pattern, and to rename it “enhanced variability gait pattern”, because of the large amount of overlapping characteristics, especially within spatio-temporal parameters.

Motor fatigability, first identified as a specific gait pattern, was dropped as a self-contained pattern. This was due to the extensive overlap between the characteristics of the motor fatigability gait pattern and the “foot drop” and “insufficient push-off” gait patterns. Motor fatigability was added as a precipitating factor, together with gait speed. This was done because in PwMS these factors can impact and influence multiple gait patterns. The level of impact on each pattern differs per patient, so it was hard to include these as separate gait patterns.

The specific “spastic” gait pattern was also removed. Defining the key characteristics of such a spastic gait pattern appeared very dependent on the muscles affected by spasticity, hence the pattern was not considered specific enough. Furthermore, the effect of spasticity of most muscles could be included in 1 of the other gait patterns (e.g., spasticity of the m. rectus femoris in the stiff knee pattern, and spasticity of m. soleus in the knee hyperextension pattern).

To summarize, the final 6 gait patterns resulting from Delphi round II were: (*i*) drop foot pattern; (*ii*) stiff knee pattern; (*iii*) knee flexion pattern; (*iv*) knee hyperextension pattern; (*v*) insufficient push-off pattern; and (*vi*) enhanced variability pattern.

### Round III (aim, methods, and results)

Round III aimed to gain further feedback on the refined gait patterns, including all characteristics and causes. Therefore, the 6 gait patterns were further specified into: “key characteristics”, “additional characteristics”, “compensatory characteristics”, “key potential cause(s)”, and “additional potential cause(s)”. Key characteristics were considered an essential aspect of a gait pattern. Additional characteristics were defined as aspects that could also be observed, but are not mandatory for a gait pattern, and may also be dependent on compensatory characteristics. Compensatory characteristics can be used to compensate for the key (and additional) characteristics. Not all compensatory characteristics are/have to be present. Similarly, key potential causes were considered specific to a gait pattern, whereas additional potential causes can also play a role in a specific gait pattern but are not necessarily present. Answers consisted of: “I agree with these characteristics” or “I do not agree with these characteristics, because”, with an open text option to explain.

The response rate of the third round was 75% (n = 15), with only 1 participant not participating in 2 consecutive rounds. Consensus on the key characteristics, additional characteristics, and key potential causes was reached for all 6 gait patterns. For compensatory characteristics and additional potential causes, consensus was attained for all gait patterns, except for the knee flexion gait pattern. For the complete consensus rates see Table III.

**Table III:** Consensus rates for the final 6 gait patterns in multiple sclerosis, including key characteristics, additional characteristics, compensatory characteristics, key potential causes, and additional potential causes

Pattern	Key characteristics	Additional characteristics	Compensatory characteristics	Key potential cause(s)	Additional potential cause(s)
Drop foot	69%	77%	85%	92%	69%
Stiff knee	75%	85%	92%	69%	75%
Knee flexion	67%	83%	58%	75%	86%
Knee hyperextension	83%	92%	83%	83%	67%
Insufficient push-off	100%	75%	92%	92%	75%
Enhanced variability	91%	80%	82%	91%	–

**Fig. 1:**
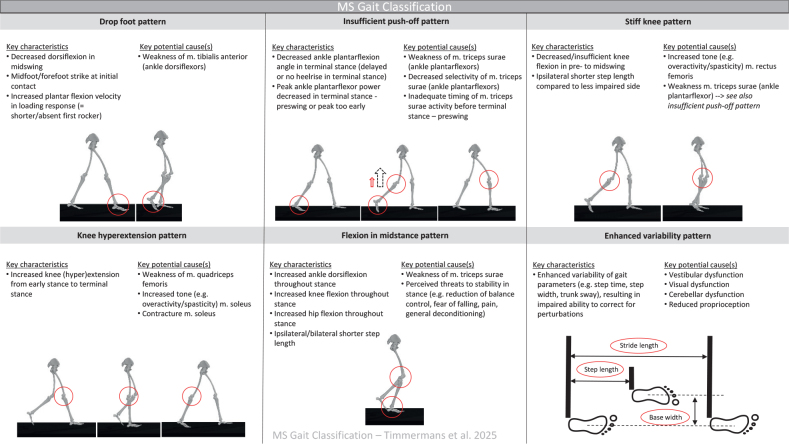
Key characteristics and key potential causes for all 6 gait patterns in people with multiple sclerosis.

The key characteristics and key potential causes can be seen in Fig. 1. The full list of characteristics and causes with consensus for the final 6 gait patterns are presented in Figs S1–S6.

Based on the results of round III, the following key characteristics were defined: (*i*) drop foot pattern with decreased dorsiflexion in mid-swing and a mid- or forefoot strike at initial contact; (*ii*) insufficient push-off pattern with decreased ankle plantarflexion in terminal stance – pre-swing and a decreased push-off power peak; (*iii*) stiff knee pattern with decreased knee flexion in pre- to mid-swing and an ipsilateral shorter step length; (*iv*) knee hyperextension pattern with increased knee (hyper-)extension from early to terminal stance; (*v*) knee flexion in midstance pattern with increased hip and knee flexion and ankle dorsiflexion throughout stance, and shorter step length; and finally (*vi*) enhanced variability pattern with enhanced variability of gait parameters, resulting in insufficient ability to correct for perturbations.

## DISCUSSION

Using a Delphi study method, the research team together with a panel of 20 international experts identified 6 key gait patterns in PwMS, including potential underlying causes. Consensus was reached on all characteristics of each gait pattern and their potential underlying causes, except for compensatory characteristics for the knee flexion in midstance pattern.

The purpose of using a gait pattern classification is to better understand, communicate about, diagnose, and treat gait disorders. In other neurological groups, especially stroke and Parkinson’s disease, specific gait patterns have been described (16, 17, 19, 29, 30). In stroke patients, terms such as hemiplegic gait, hemiparetic gait, and spastic gait have been used (17), while in patients with Parkinson’s disease freezing, stooped posture, shuffling steps, and falling are seen as typical (16). In PwMS, Filli et al. identified 3 pathological gait patterns; spastic-paretic, ataxia-like, and unstable gait (29), while Kempen et al. defined 3 distinct gait classes, which were clearly related to the progressive course of MS, seemingly induced by increasing insufficiency of the ankle push-off and tibialis anterior muscle weakness (30). Alongside muscle weakness, impaired coordination, balance, sensation, or spasticity can also play a role. MS is heterogeneous in its clinical presentation, leading to a wide variety of possible deviations of gait characteristics and therefore a wide variety of possible patterns. This is partly due to the fact that multiple neurological systems that influence gait can be impacted in PwMS, e.g., pyramidal, cerebellar, sensory, visual. Yet common themes and characteristics can be observed, partly due to different phases of MS disease progression and partly due to lesion location (cortical, cerebellar, or spinal cord). Supplementing the previously mentioned studies, the current study helped to combine the common themes and characteristics into patterns, aiding in our understanding, communication, and clinical care of gait disorders in PwMS.

A difference in use of terminology between the different occupational groups was observed in the responses. Some physiotherapists preferred to use terms such as “hip hiking”, “vaulting”, and “foot flat”. In contrast, movement scientists and rehabilitation physicians preferred kinematic terminology such as “reduced (ankle) dorsiflexion at initial contact” or “reduced hip flexion in mid-swing”. Supporting our observations, a similar difference in terminology has been described by Watelain et al., who found that physiotherapists were highly descriptive, rehabilitation physicians took a more biomechanical approach, and neurologists focused on elements to help identify lesion localization (31). However, the use of different terminology can hinder cooperation and communication between different healthcare professionals. Therefore, we opted to convert all answers to consistent kinematic terminology. Kinematic terminology is used worldwide and was recommended in 1990 by the International Society of Biomechanics (28). In round II, consensus rates were low. The introduction of the kinematic terminology and answers of the participants in round III substantially improved consensus. This supports the use of standardized nomenclature to enhance interdisciplinary collaboration and communication.

The Delphi design was an efficient way of gathering international expertise within a relatively short time period. Generally, a Delphi study starts with all information already gathered and many questions, and works to eliminate or accept specific information through consensus. By starting the study with 2 open questions we gathered as much information as possible, without influencing the input of the participants too much. The answers from round I were already very complete concerning the gait patterns and impairments that were responsible for certain gait patterns. Earlier research indicated that 3 Delphi rounds are often enough to gather the information and to reach consensus (32). Indeed, in our study, we used 3 rounds, leading to consensus on most items. In Delphi studies, wide consensus rates between 50% and 97% are described in the literature (33). In this study we used 67% as consensus criterion, i.e., two-thirds of the participants had to agree. A higher consensus rate would have resulted in less agreement on multiple characteristics, as various characteristics now had a consensus of 67% or 69%. Consensus on naming of the gait patterns was 73% and above.

For the definition of the gait patterns, we adopted a classification based on the most prominent feature of the gait pattern (e.g., a “dropped foot” is the most prominent feature of the “drop foot” pattern). This choice was made because in our opinion it most accurately encapsulates the answers of the experts, and portrays the clinical situation, while also offering starting points for treatment. Other classification ideas were also considered, such as (*i*) a “classic” classification, using hemiparetic, spastic, and ataxic; (*ii*) a classification based on “signs”, with a differential diagnosis for each sign in MS (34); (*iii*) a classification based on joints (e.g., trunk, pelvis, ankle etc.); and (*iv*) a classification based on global terms (e.g., weakness, hypertonia, imbalance, etc.). The often mentioned “hemiparetic gait” was ultimately not included as a specific gait pattern, as this pattern showed clear overlap with other gait patterns.

The resulting 6 identified gait patterns specific for gait disorders in PwMS can assist healthcare professionals in multiple ways. First, they can be used as a tool in the communication between healthcare providers by unifying terminology. Second, they can support communication between healthcare providers and patients by providing insight into the underlying gait problem experienced by a patient. Third, they can help in monitoring within-patient changes in gait over time more precisely. Finally, they can aid in treatment decisions. To serve this latter purpose, this study will be continued with 3 subsequent rounds with the same sample of participants, to establish consensus regarding diagnostics for the potential underlying causes of the gait patterns, and to identify best practice treatment options for each of the 6 gait patterns. A future follow-up paper will focus on these outcomes regarding diagnostics and treatment. Based on all results, we plan to develop a clinical decision tree, to help with decision-making on gait disorders in PwMS in daily clinical practice. This will enable healthcare centres and practices to decide more easily between treatment options, and potentially refer to a specialized MS centre when needed.

### Strengths and limitations

A strength of this study is the worldwide distribution of its participants, including experience and expert opinion from a wide range of international practices. Also, a broad range of experts with experience with MS patients in both clinical and research areas participated.

The gait patterns were derived by content analysis performed by the first author with regular direct feedback from the entire research team. This could have potentially introduced some bias in interpretation of the expert opinions. The research team had an impact on the study by analysing, dividing, encapsulating, and resubmitting the answers of the participants. However, the research team was, with its clinical and research expertise, a good reflection of the group of collaborators in the Delphi study. Therefore, we believe that the research team was well qualified to weigh all input and make well-considered decisions. The eventual bias in interpretation was mitigated as much as possible by the collective consensus of the gait patterns, underlying characteristics, and potential causes, and the opportunity for participants to correct any misrepresentation or omission in subsequent Delphi rounds. Due to the heterogeneity of MS and the nature of a Delphi study it is possible that the Delphi process did not capture all gait impairments seen in individual MS patients. It is also possible that less common gait patterns, which were currently excluded, could still be clinically relevant in a smaller group of patients requiring intervention.

The nature of a Delphi study, leading to consensus between experts using a qualitative research method, does not necessarily lead to the correct answers, but to the most supported answers. This was partly mitigated by the experience in daily healthcare practice and research of the participating group. All participants, as well as the clinical and research MS community, will be facilitated and are encouraged to use the results in their own clinical practice and research groups. Further study is recommended using quantitative data to confirm the current Delphi results, for example using gait analysis data, or other data from daily clinical care.

In conclusion, relevant consensus on 6 gait patterns in patients with MS with their key characteristics and potential causes was reached. The standardization of these gait patterns in MS will improve the understanding of impairments that underlie gait problems in PwMS, and support clinical decision-making and communication regarding diagnosing and treatment of these gait problems.

## Supplementary Material

A DELPHI STUDY TO IDENTIFY KEY GAIT PATTERNS AND THEIR POTENTIAL CAUSES IN PEOPLE WITH MULTIPLE SCLEROSIS

A DELPHI STUDY TO IDENTIFY KEY GAIT PATTERNS AND THEIR POTENTIAL CAUSES IN PEOPLE WITH MULTIPLE SCLEROSIS

## References

[1] Kister I, Bacon TE, Chamot E, Salter AR, Cutter GR, Kalina JT, Herbert J. Natural history of multiple sclerosis symptoms. Int J MS Care 2013; 15: 146–158. 10.7224/1537-2073.2012-053 24453777 PMC3883021

[2] Timmermans ST, de Groot V, Beckerman H. Ten-year disease progression in multiple sclerosis: walking declines more rapidly than arm and hand function. Mult Scler Relat Disord 2020; 45: 102343. 10.1016/j.msard.2020.102343 32674028

[3] Sutliff MH. Contribution of impaired mobility to patient burden in multiple sclerosis. Curr Med Res Opin 2010; 26: 109–119. 10.1185/03007990903433528 19916707

[4] Bethoux F. Gait disorders in multiple sclerosis. Continuum (Minneap Minn) 2013; 19: 1007–1022. 10.1212/01.CON.0000433286.92596.d5 23917098

[5] Eken MM, Richards R, Beckerman H, van der Krogt M, Gerrits K, Rietberg M, et al. Quantifying muscle fatigue during walking in people with multiple sclerosis. Clin Biomech (Bristol) 2020; 72: 94–101. 10.1016/j.clinbiomech.2019.11.020 31862607

[6] Galea MP, Cofre Lizama LE, Butzkueven H, Kilpatrick TJ. Gait and balance deterioration over a 12-month period in multiple sclerosis patients with EDSS scores </= 3.0. NeuroRehabilitation 2017; 40: 277–284. 10.3233/NRE-161413 28222549

[7] Scholz M, Haase R, Trentzsch K, Weidemann ML, Ziemssen T. Fear of falling and falls in people with multiple sclerosis: Aa literature review. Mult Scler Relat Disord 2021; 47: 102609. 10.1016/j.msard.2020.102609 33189021

[8] Bennett SE, Bromley LE, Fisher NM, Tomita MR, Niewczyk P. Validity and reliability of four clinical gait measures in patients with multiple sclerosis. Int J MS Care 2017; 19: 247–252. 10.7224/1537-2073.2015-006 29070965 PMC5649348

[9] Lizrova Preiningerova J, Novotna K, Rusz J, Sucha L, Ruzicka E, Havrdova E. Spatial and temporal characteristics of gait as outcome measures in multiple sclerosis (EDSS 0 to 6.5). J Neuroeng Rehabil 2015; 12: 14. 10.1186/s12984-015-0001-0 25890382 PMC4334845

[10] Shanahan CJ, Boonstra FMC, Cofre Lizama LE, Strik M, Moffat BA, Khan F, et al. Technologies for advanced gait and balance assessments in people with multiple sclerosis. Front Neurol 2017; 8: 708. 10.3389/fneur.2017.00708 29449825 PMC5799707

[11] Hobart JC, Riazi A, Lamping DL, Fitzpatrick R, Thompson AJ. Measuring the impact of MS on walking ability: the 12-Item MS Walking Scale (MSWS-12). Neurology 2003; 60: 31–36. 10.1212/wnl.60.1.31 12525714

[12] Sosnoff JJ, Sandroff BM, Motl RW. Quantifying gait abnormalities in persons with multiple sclerosis with minimal disability. Gait Posture 2012; 36: 154–156. 10.1016/j.gaitpost.2011.11.027 22424761

[13] Coca-Tapia M, Cuesta-Gomez A, Molina-Rueda F, Carratala-Tejada M. Gait pattern in people with multiple sclerosis: a systematic review. Diagnostics 2021; 11; 584. 10.3390/diagnostics11040584 33805095 PMC8064080

[14] Comber L, Galvin R, Coote S. Gait deficits in people with multiple sclerosis: a systematic review and meta-analysis. Gait Posture 2017; 51: 25–35. 10.1016/j.gaitpost.2016.09.026 27693958

[15] Becher JG. Pediatric rehabilitation in children with cerebral palsy: general management, classification of motor disorders. JPO: Journal of Prosthetics and Orthotics 2002; 14.4: 143–149. 10.1097/00008526-200212000-00004

[16] Chen PH, Wang RL, Liou DJ, Shaw JS. Gait disorders in Parkinson’s disease: assessment and management. Int J Gerontol 2013; 7: 189–193. 10.1016/j.ijge.2013.03.005

[17] Li S, Francisco GE, Zhou P. Post-stroke hemiplegic gait: new perspective and insights. Front Physiol 2018; 9: 1021. 10.3389/fphys.2018.01021 30127749 PMC6088193

[18] Rodda J, Graham HK. Classification of gait patterns in spastic hemiplegia and spastic diplegia: a basis for a management algorithm. Eur J Neurol 2001; 8 Suppl 5: 98–108. 10.1046/j.1468-1331.2001.00042.x 11851738

[19] Snijders AH, van de Warrenburg BP, Giladi N, Bloem BR. Neurological gait disorders in elderly people: clinical approach and classification. Lancet Neurol 2007; 6: 63–74. 10.1016/S1474-4422(06)70678-0 17166803

[20] Winters TF Jr, Gage JR, Hicks R. Gait patterns in spastic hemiplegia in children and young adults. J Bone Joint Surg Am 1987; 69: 437–441. 10.2106/00004623-198769030-00016 3818706

[21] Hasson F, Keeney S, McKenna H. Research guidelines for the Delphi survey technique. J Adv Nurs 2000; 32: 1008–1015. 10.1046/j.1365-2648.2000.t01-1-01567.x 11095242

[22] Haven TL, Errington TM, Gleditsch KS, van Grootel L, Jacobs AM, Kern FG, et al. Preregistering qualitative research: a Delphi study. Int J Qual Meth 2020; 19. 10.1177/1609406920976417

[23] Okoli C, Pawlowski SD. The Delphi method as a research tool: an example, design considerations and applications. Inform Manage-Amster 2004; 42: 15–29. 10.1016/j.im.2003.11.002

[24] Akins RB, Tolson H, Cole BR. Stability of response characteristics of a Delphi panel: application of bootstrap data expansion. BMC Med Res Methodol 2005; 5: 37. 10.1186/1471-2288-5-37 16321161 PMC1318466

[25] Nair R, Aggarwal R, Khanna D. Methods of formal consensus in classification/diagnostic criteria and guideline development. Semin Arthritis Rheum 2011; 41: 95–105. 10.1016/j.semarthrit.2010.12.001 21420149 PMC3131416

[26] Waggoner J, Carline JD, Durning SJ. Is there a consensus on consensus methodology? Descriptions and recommendations for future consensus research. Acad Med 2016; 91: 663–668. 10.1097/ACM.0000000000001092 26796090

[27] Terwee CB, Prinsen CAC, Chiarotto A, Westerman MJ, Patrick DL, Alonso J, et al. COSMIN methodology for evaluating the content validity of patient-reported outcome measures: a Delphi study. Qual Life Res 2018; 27: 1159–1170. 10.1007/s11136-018-1829-0 29550964 PMC5891557

[28] Wu G, Cavanagh PR. ISB recommendations for standardization in the reporting of kinematic data. J Biomech 1995; 28: 1257–1261. 10.1016/0021-9290(95)00017-c 8550644

[29] Filli L, Sutter T, Easthope CS, Killeen T, Meyer C, Reuter K, et al. Profiling walking dysfunction in multiple sclerosis: characterisation, classification and progression over time. Sci Rep 2018; 8: 4984. 10.1038/s41598-018-22676-0 29563533 PMC5862880

[30] Kempen JC, Doorenbosch CA, Knol DL, de Groot V, Beckerman H. Newly identified gait patterns in patients with multiple sclerosis may be related to push-off quality. Phys Ther 2016; 96: 1744–1752. 10.2522/ptj.20150508 27174257

[31] Watelain E, Froger J, Barbier F, Lensel G, Rousseaux M, Lepoutre FX, et al. Comparison of clinical gait analysis strategies by French neurologists, physiatrists and physiotherapists. J Rehabil Med 2003; 35: 8–14. 10.1080/16501970306104 12610842

[32] Hsu C-C, Brian A. The Delphi Technique: making sense of consensus. Practical Assessment, Research, and Evaluation 2007; 12: 10. 10.7275/pdz9-th90

[33] Diamond IR, Grant RC, Feldman BM, Pencharz PB, Ling SC, Moore AM, et al. Defining consensus: a systematic review recommends methodologic criteria for reporting of Delphi studies. J Clin Epidemiol 2014; 67: 401–409. 10.1016/j.jclinepi.2013.12.002 24581294

[34] Nonnekes J, Goselink RJM, Ruzicka E, Fasano A, Nutt JG, Bloem BR. Neurological disorders of gait, balance and posture: a sign-based approach. Nat Rev Neurol 2018; 14: 183–189. 10.1038/nrneurol.2017.178 29377011

