# Supplementary material for: A DELPHI STUDY TO IDENTIFY KEY GAIT PATTERNS AND THEIR POTENTIAL CAUSES IN PEOPLE WITH MULTIPLE SCLEROSIS
Source: J Rehabil Med. 2025 Jun 3;57:42556. doi: 10.2340/jrm.v57.42556 (PMC12159873; doi:10.2340/jrm.v57.42556)
Supplement: A DELPHI STUDY TO IDENTIFY KEY GAIT PATTERNS AND THEIR POTENTIAL CAUSES IN PEOPLE WITH MULTIPLE SCLEROSIS [file JRM-57-42556-s1.pdf]

Supplementary material has been published as submitted. It has not been copyedited, or typeset by Journal of Rehabilitation Medicine

APPENDIX S1

Supplementary material – round specific instructions

Delphi instructions round I

Welcome to the first round of the Delphi study on "clinical decision making of gait disorders in Multiple Sclerosis".

Main question: Which gait features and impairments contribute to gait disorders in MS patients?

The **primary objective** of this study is to develop a clinical decision tree, relating gait features and impairments to the different diagnostic and treatment options to facilitate clinical decision making in MS patients with gait disorders.

The focus will be on generalization, not on one specific patient or diagnostic and/or treatment outliers.

In this round, possible diagnostics and treatment options are NOT yet discussed (these will be added in round 3 and 5 respectively).

Instructions:
Please provide **as many answers** (and if possible, explanations) that apply to the provided question. The aim is to gather a list of gait features and impairments as extensive as possible.

<u>Delphi instructions round II</u>

Welcome to the **second** round of the Delphi study on "clinical decision making of gait disorders in Multiple Sclerosis".

This round will take you about 30 min to 45 min, depending on how much you agree with.

<u>Main question</u>: Which gait features and impairments contribute to gait disorders in MS patients?

We gathered all your answers and used them to **group** features to create gait patterns that (often) appear in patients with Multiple Sclerosis. The 'created' patterns are named in bold letters (e.g. drop foot pattern).

<u>Instructions</u>:
- On every page you'll have to answer 4 questions concerning:
1) The naming of the pattern
2) Kinematic and Kinetic deviations of this pattern
3) Spatiotemporal deviations of this pattern
4) Causes of this pattern

- Please answer every question. The aim is to get **consensus** on the list of gait features and impairments gathered in the first round and to get consensus on patterns and their naming, i.e. combination of features and impairments.

<u>Please note that the presented patterns can occur together and that mixed patterns often appear.</u>

<u>Delphi instructions round III</u>

Dear Delphi participants,

Thank you all so much for your input. We used all of your answers to change and adapt the gait patterns when necessary. [see link for figure of consensus]

To be able to better unify the given answers we opted for the use of pre-existing **kinematic and kinetic gait terminology**. So after sorting all the information we 'translated' your answers, when applicable, to a model of kinematic and kinetic terminology (e.g. 'increased plantar flexion velocity in loading response' instead of 'foot flat'). We intended to use terminology that can be **universally understood** and describes the gait feature as **clearly as possible**.

The biggest changes applied are a reduction in the number of gait patterns and the layout within the patterns itself.

<u>Reduction of number of gait patterns:</u>
Based on your answers/feedback we decided to **combine** the '*somatosensory*' and '*reduced balance control*' patterns with the '*ataxic*' pattern to '*enhanced variability pattern*,' because of the large amount of overlapping characteristics. The '*motor fatigability*' pattern was **removed** and was incorporated in the introduction section (see below; precipitating factors).
A specific '*spastic*' pattern was removed, as the characteristics are very dependent on the muscle affected. Furthermore, most affected muscles could be sorted in the other gait patterns.

<u>Layout change of patterns:</u>
Furthermore, we decided to **split characteristics** into 'key defining characteristics' and 'additional characteristics' to put more focus on the essential features. This also applies to the causes, which we split into 'key causes' and 'additional causes'. Key causes are (almost) always seen with that pattern. Additional causes *can* be seen with a pattern, but do not define it. We also added a **separate compensatory section** for every pattern.

<u>General influencing factors:</u>
There are some influencing factors that (can) **impact and influence multiple gait patterns** in MS. The amount of impact on the patient differs, so it's hard to include these in the separate patterns. These factors consist of **motor fatigability** and **gait speed**.

<u>Delphi round 3:</u>
Main question of this third Delphi round: *Which **diagnostics** can be used to determine specific gait features and impairments that contribute to gait disorders in MS patients?*

The first part of questions of Round 3 concerns the changes we made using your answers from Round 2 (do you agree, or not; and why not). To focus on the main question of this Round 3 (**diagnostics**), this part is rather short. The second part of questions concerns the diagnostics for the specific impairments.

<u>Information and instructions for Round 3</u>:
There is one pattern per page; divided in: "key characteristics," "additional characteristics," "compensatory characteristics," "key potential cause(s)" and "additional potential cause(s)."
- The possible answers are: "I agree, or I do not agree; and if not, why not"

This is followed by a matrix question focusing on the diagnostics that can be used to diagnose the key and additional potential cause(s).
- You can click on all diagnostic options you use, or would use if available to you. Use the slider to see all answers.

- Try to answer every question.
