# Supplementary material for: A DELPHI STUDY TO IDENTIFY KEY GAIT PATTERNS AND THEIR POTENTIAL CAUSES IN PEOPLE WITH MULTIPLE SCLEROSIS
Source: J Rehabil Med. 2025 Jun 3;57:42556. doi: 10.2340/jrm.v57.42556 (PMC12159873; doi:10.2340/jrm.v57.42556)
Supplement: A DELPHI STUDY TO IDENTIFY KEY GAIT PATTERNS AND THEIR POTENTIAL CAUSES IN PEOPLE WITH MULTIPLE SCLEROSIS [file JRM-57-42556-s2.pdf]

Supplementary material has been published as submitted. It has not been copyedited, or typeset by Journal of Rehabilitation Medicine

Fig. S1. All characteristics and causes for the drop foot pattern in people with multiple sclerosis.

Drop foot pattern

Key characteristics
- Decreased dorsiflexion in midswing
- Midfoot/forefoot strike at initial contact
- Increased plantar flexion velocity in loading response (= shorter/absent first rocker)

Additional characteristics
- Ipsilateral shorter step length compared to less affected side
- Foot/ankle inversion in mid and terminal swing

Compensatory characteristics
- Increased knee flexion in mid swing
- Increased hip flexion in midswing
- Increased hip abduction (and exorotation) in midswing (= circumduction)
- Ipsilateral pelvic lift in midswing
- Contralateral heelrise in midstance (= vaulting)

Key potential cause(s)
- Weakness of m. tibialis anterior (ankle dorsiflexors)

Additional potential cause(s)
- Weakness m. peroneus tertius
- Weakness toe extensors
- Weakness m. peroneus longus (foot evertor) for inversion
- Contracture m. tibialis posterior
- Increased tone (e.g. overactivity/spasticity) m. tibialis posterior
- Increased tone (e.g. overactivity/spasticity) in m. triceps surae
- Contracture of m. triceps surae

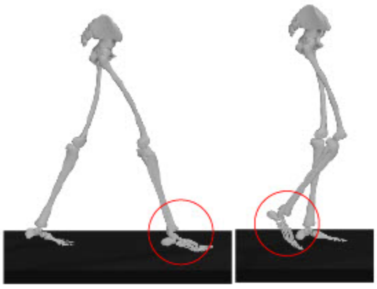

MS Gait Classification – Timmermans et al.

Fig. S2. All characteristics and causes for the insufficient push-off pattern in people with multiple sclerosis.

Insufficient push-off pattern

Key characteristics
- Decreased ankle plantarflexion angle in terminal stance (delayed or no heelrise in terminal stance)
- Peak ankle plantarflexor power decreased in terminal stance - preswing or peak too early

Additional characteristics
- Reduced clearance in initial swing
- Increased knee flexion in terminal stance
- Decreased knee flexion in midswing (unless compensated by knee flexors)
- Decreased hip flexion in midswing (unless compensated by hipflexors)
- Decreased knee extension in terminal swing
- Mid/forefoot landing at initial contact
- Ipsilateral shorter step length

Compensatory characteristics
- Increased generation of power at hip level throughout swing
- Increased generation of hip power in loading response early stance; ipsi/contralateral

Key potential cause(s)
- Weakness of m. triceps surae (ankle plantarflexors)
- Decreased selectivity of m. triceps surae (ankle plantarflexors)
- Inadequate timing of m. triceps surae activity before terminal stance – preswing

Additional potential cause(s)
- Contracture of triceps surae
- Increased tone (e.g. overactivity/spasticity) of triceps surae during stance
- Co-activation of ankle dorsiflexors and plantarflexors

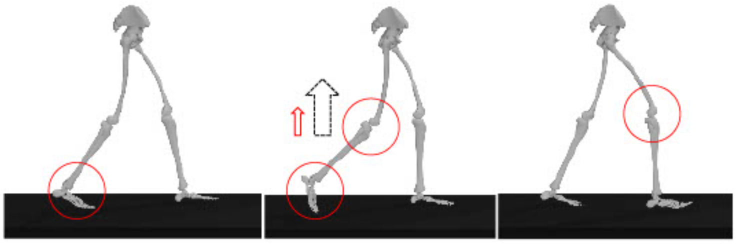

MS Gait Classification – Timmermans et al.

Fig. S3. All characteristics and causes for the stiff knee pattern in people with multiple sclerosis.

Stiff knee pattern

Key characteristics
- Decreased/insufficient knee flexion in pre- to midswing
- Ipsilateral shorter step length compared to less impaired side

Additional characteristics
- Decreased knee extension in late swing
- Reduced hip power and work in preswing and early swing (lack off pull-off
- mechanism at hip level)
- Reduced foot clearance in swing
- Increased step width (wider base of support)
- Increased double support time

Compensatory characteristics
- Increased hip abduction (and exorotation) in midswing (= circumduction)
- Contralateral lateroflexion of the trunk in midswing combined with ipsilateral pelvic lift
- Contralateral heelrise in midstance (vaulting)
- Increased hip flexion in midswing
- Increased ankle dorsalflexion in midswing

Key potential cause(s)
- Increased tone (e.g. overactivity/spasticity) m. rectus femoris
- Weakness m. triceps surae (ankle plantarflexor) --> *see also insufficient push-off pattern*

Additional potential cause(s)
- Contracture m. rectus femoris
- Weakness of the hip flexors (iliopsoas)
- Reduction of lower limb control and interlimb coordination (co-activation)
- Knee joint related (arthritis, pain, swelling)

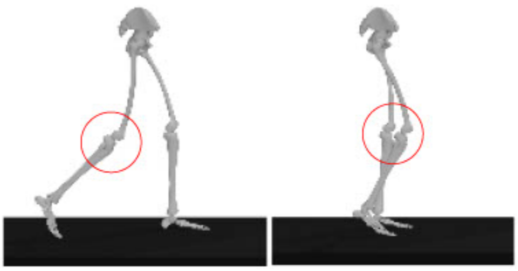

MS Gait Classification – Timmermans et al.

Fig. S4. All characteristics and causes for the knee hyperextension pattern in people with multiple sclerosis.

Knee hyperextension pattern

Key characteristics
- Increased knee (hyper)extension from early stance to terminal stance

Additional characteristics
- Increased knee extension from early stance (if weakness is the main cause)
- Decreased ankle dorsal flexion in midstance
- Reduced contralateral step length
- Increased knee extension (hyperextension) during preswing

Compensatory characteristics
- Increased hip flexion throughout stance
- Increased pelvic anterior tilt throughout stance
- Trunk anteflexion throughout stance
- Increased pelvic anterior (or posterior) tilt

Key potential cause(s)
- Weakness of m. quadriceps femoris
- Increased tone (e.g. overactivity/spasticity) m. soleus
- Contracture m. soleus

Additional potential cause(s)
- Full lower limp incoordination (especially loss of knee coordination/decreased knee proprioception)

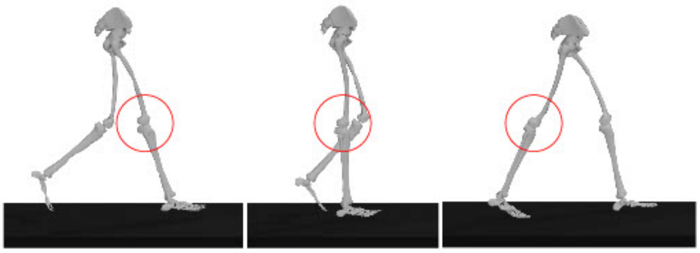

MS Gait Classification – Timmermans et al.

Fig. S5. All characteristics and causes for the flexion in midstance pattern in people with multiple sclerosis.

Flexion in midstance pattern

Key characteristics
- Increased ankle dorsiflexion throughout stance
- Increased knee flexion throughout stance
- Increased hip flexion throughout stance
- Ipsilateral/bilateral shorter step length

Additional characteristics
- Decreased hip extension in terminal stance
- Increased pelvic anterior tilt throughout stance
- Forward lean of trunk
- Loss of force absorption in loading response in the knee joint
- Push-off leg less posterior in relation to stance leg, resulting in a more vertical than horizontal push-off
- Increased contralateral trunk sway throughout stance

Compensatory characteristics
- -

Key potential cause(s)
- Weakness of m. triceps surae
- Perceived threats to stability in stance (e.g. reduction of balance control, fear of falling, pain, general deconditioning)

Additional potential cause(s)
- Contracture of hip flexors
- Increased tone (e.g. overactivity/spasticity) of hip flexors
- Contracture of hamstrings
- Increased tone (e.g. overactivity/spasticity) of hamstrings
- Contracture of m. gastrocnemius
- Increased tone (e.g. overactivity/spasticity) of m. gastrocnemius
- Loss of knee coordination/decreased knee propriocepsis
- Weakness of hip extensors

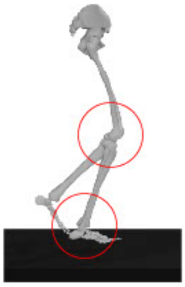

MS Gait Classification – Timmermans et al.

Fig. S6. All characteristics and causes for the enhanced variability pattern in people with multiple sclerosis.

Enhanced variability pattern

Key characteristics
- Enhanced variability of gait parameters (e.g. step time, step width, trunk sway), resulting in impaired ability to correct for perturbations

Additional characteristics
- Difficulty with pertubations/turning
- Increased double support time
- Decreased anterior-posterior and/or increased medio-lateral trunk sway
- Increased center of pressure sway of stance leg (reduced stability)
- Increased anterior-posterior and medio-lateral margins of stability (MoS)
- Forward lean (to increase visual feedback)

Compensatory characteristics
- Increased knee flexion throughout stance (from early to terminal stance)
- Increased hip flexion throughout stance

Key potential cause(s)
- Vestibular dysfunction
- Visual dysfunction
- Cerebellar dysfunction
- Reduced proprioception

Additional potential cause(s)
- None

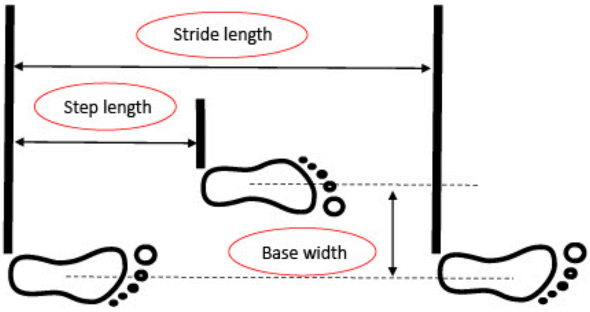

MS Gait Classification – Timmermans et al.
